# A Case Report of Lupus Cerebritis in a Female With Preceding Kikuchi-Fujimoto Disease

**DOI:** 10.7759/cureus.82594

**Published:** 2025-04-19

**Authors:** Hussein A Al Barazanjy, Ali R Alabdullah, Azhar Shabbir, Mohammed Q Raheemah AlMalchi, Ameer F Hussein

**Affiliations:** 1 Neurology, Al-Najaf Al-Ashraf Teaching Hospital, Al-Najaf, IRQ; 2 Neurology, Al-Najaf Al-Ashraf Teaching Hospital, Poiliclinico San Donato, Al-Najaf, IRQ; 3 General Surgery, Al-Najaf Al-Ashraf Teaching Hospital, Poiliclinico San Donato, Al-Najaf, IRQ; 4 General Surgery, Hamad Medical Corporation, Doha, QAT

**Keywords:** antinuclear antibodies, cervical lymphadenopathy, kikuchi fujimoto illness, male, systemic lupus erythematosus

## Abstract

A rare, benign, self-limiting illness known as Kikuchi-Fujimoto disease (KFD) is characterized by fever and lymphadenopathy in young females. Systemic lupus erythematosus (SLE), on the other hand, is a fairly prevalent autoimmune disease. Kikuchi disease is sometimes associated with SLE, with which it may coexist. To validate the diagnosis, the presence of necrotizing lymphadenitis is considered to be of significance. A positive anti-nuclear antibody (ANA) suggests a potential association with SLE or a relapse of the underlying condition. To avoid incorrect diagnosis and ineffective therapy, this clinical presentation requires a comprehensive evaluation. Steroids and immunological therapy are used to treat Kikuchi illness, which is a persistent and recurrent condition that usually requires supportive care. Early diagnosis of ominous disorders requires long-term surveillance.

## Introduction

Kikuchi-Fujimoto Disease (KFD), also known as Kikuchi disease or histiocytic necrotizing lymphadenitis, was first described in Japan and remains a rare but recognized entity worldwide [1]. It typically presents with fever and cervical lymphadenopathy, although the clinical spectrum can vary [2]. Historically, KFD was thought to show a female predominance, but more recent epidemiological data suggest that the gender distribution may be closer to equal [1-3]. While the precise pathogenesis remains unclear, the disease is believed to involve a T-cell-mediated immune response, potentially triggered by an underlying infection. This immune dysregulation could explain its documented links to autoimmune disorders, including but not limited to systemic lupus erythematosus (SLE). Associations with infectious triggers have also been reported [3].

The relationship between KFD and SLE has attracted particular attention in the literature, with studies indicating that KFD precedes the onset of SLE in roughly 30% of cases, while in about 23% of cases, KFD develops after the diagnosis of SLE [4]. SLE itself is a chronic autoimmune disorder with multi-system involvement and a strong predilection for females of reproductive age. This gender disparity is believed to result from a combination of hormonal factors (such as estrogen), environmental exposures, and genetic predisposition related to the X chromosome. Epidemiological data estimate a female-to-male ratio between 5:1 and 7:1 [5]. Interestingly, male-onset SLE tends to occur later in life, with an average age of presentation around 40 years [5].

In this report, we describe the case of a previously healthy adolescent female, who was diagnosed with biopsy-confirmed Kikuchi-Fujimoto Disease, later followed by the development of systemic lupus erythematosus. This case highlights the importance of recognizing KFD as a possible early marker of evolving systemic autoimmunity, emphasizing the need for ongoing surveillance in such patients.

## Case presentation

A 23-year-old female with no significant past medical history presented with symptoms that began approximately seven months ago. Initially, she noticed a lump on the left side of her neck, approximately 1x1 cm in size, round in shape, with a smooth surface. The lump was painless, not warm to the touch, soft in consistency, and mobile under the skin. It fluctuated in size during the first two months but progressively increased afterward. A week later, she developed an intermittent fever, occasionally with two spikes per day, though not documented by a thermometer. This fever was not associated with rigors or chills but was accompanied by varying episodes of night sweats. These symptoms occurred on some days and subsided on others, but persisted overall. The patient also reported a lack of appetite.

A month into her symptoms, she noticed multiple lumps in both her right and left armpits, which presented in the same manner as the neck lump. She sought medical advice at a surgical clinic, where a biopsy of one of the lumps was recommended, the results of which were communicated as changes consistent with reactive lymphoid tissue with no other significant findings, and she received medication to control her symptoms only. This led to a partial improvement, as her fever subsided for two weeks but eventually returned and remained intermittent. Two months later, she began experiencing progressive difficulty in walking. Initially, this was characterized by her left foot slipping out of her shoe and lurching to the left side. She also noted difficulty climbing stairs. Over time, this progressed to difficulty standing after sitting, and in the last month, she became unable to walk unaided, developing unsteadiness and lurching to both sides.

During this period, she also began to experience difficulty using her left hand. She found it hard to hold objects, leading to frequent slipping, and she struggled with tasks such as buttoning her clothes. A month before her presentation to our department, she developed urinary incontinence, described as the insensible passage of urine. Three weeks later, she was advised to undergo a second lump biopsy. Two weeks later, her condition worsened with behavioral changes. She refused to talk to her family and chose to stay alone for extended periods. A week ago, she became increasingly unresponsive to commands and exhibited excessive daytime sleepiness.

The day before her hospital admission, she experienced abnormal body movements. These included upward rolling of her eyes and stiffening of her upper limbs, lasting three minutes, followed by a progressive return to consciousness over 45 minutes. Later the same day, she experienced a second similar episode. She reported frequent episodes of black stools over the past six months, occurring several times a month. However, these episodes were not accompanied by abdominal pain, nausea, or vomiting. She estimated a total weight loss of 14 kilograms since the onset of her symptoms.

She did not describe double vision, hearing loss, difficulty swallowing, skin rash, genital or oral ulcers, joint pain, or a family history of a similar condition.

On general examination, the patient appeared pale and fatigued but was conscious and oriented. Her build was noticeably slim due to significant weight loss. A linear scar was observed on the left side of her neck from a previous biopsy. No signs of dyspnea, tachypnea, or rash were noted. Examination of the chest and abdomen was unremarkable.

A neurological examination revealed her to be conscious and oriented, with intact cognition but impaired attention and concentration. Further examination of the cranial nerves showed bilateral disc swelling, broken eye pursuit, and an exaggerated jaw reflex. Left-sided distal muscle weakness and bilateral action tremors were observed on upper limb examination. Examination of the lower limbs revealed bilateral hypotonia, more pronounced on the left, diminished reflexes, and weakness graded 4/5 in the left and 4+/5 in the right lower limbs. Babinski’s reflex was downgoing bilaterally.

Table 1 highlights key parameters of the CSF analysis on admission, including elevated protein levels (345 mg/dl) and a positive result for globulin (Pandy’s test). The CSF was described as very slightly xanthochromic and clear, with normal levels of cells and negative results for bacterial, fungal, and other pathological screenings.

**Table 1:** Findings of the cerebrospinal fluid (CSF) analysis LDH: lactate dehydrogenase; G.M. film: Giemsa-stained blood film; Z.N.: Ziehl-Neelsen; AFB: acid-fast bacilli; VDRL: Venereal Disease Research Laboratory

Parameter	Result	Reference Range	Remarks
Color & Appearance	Very slight xanthochromic.	Clear and colorless	Slight xanthochromia is abnormal.
Specific Gravity	1.004	1.003 - 1.008	Values outside may suggest abnormalities.
Sugar	45 mg/dl	45-80 mg/dl	Low levels could indicate infection.
Protein	345 mg/dl	15-45 mg/dl	Elevated levels indicate infections or inflammation.
Globulin (Pandy's Test)	Positive	Negative	Positive indicates abnormal globulin levels.
LDH	96 u/l	Up to 40 u/l	Higher levels suggest tissue damage.
RBCs	0/cmm	0 /cmm	Presence may indicate trauma or hemorrhage.
WBCs	3/cmm	0-5 /cmm	Elevated levels may suggest infection or inflammation.
Lymphocytes	100%	100%	Should dominate in normal CSF.
Neutrophils	0%	0%	Presence indicates bacterial infection.
Monocytes	0%	0%	Usually absent in normal CSF.
G.M. Film (Bacteria)	Negative	Negative	Positive indicates bacterial infection.
Z.N. Film (AFB)	Negative	Negative	Positive indicates tuberculosis.
Rose Bengal	Negative	Negative	Positive indicates brucellosis.
VDRL	Negative	Negative	Positive indicates syphilis.
Film for Fungus	Negative	Negative	Positive indicates a fungal infection.
Pressure	21 cm of water	10-20 cm H_2_O	Elevated in infection or hemorrhage.
Supernatant Color	-	Clear and colorless	
Deposit Film	-	1.003 - 1.008	

Table 2 summarizes the results of various autoimmune markers. Anti-nuclear antibody (ANA) immunoglobulin G (IgG) and anti-smooth muscle were normal while IgG, C-ANCA (PR3), and P-ANCA (MPO) showed mild elevations while all other markers, including dsDNA, nucleosomes, and smD1, were negative, indicating the absence of significant autoimmune involvement. 

**Table 2:** Connective tissue screening laboratory findings

Test Name	Result	Unit	Reference Range
ANA IgG	0.38	Index	Negative: <1.0 Equivocal: 1.0-1.2 Positive: >1.2
Anti-Smooth Muscle IgG	1.5		Negative: <12 Equivocal: 12-18 Positive: >18
C-ANCA (PR3)	2.4	Au/ml	Negative: <2 Positive: >2
P-ANCA (MPO)	2.1	Au/ml	Negative: <2 Positive: >2
IMTEC-ANA-LIA-MAXX (Connective Tissue Screen)			
dsDNA	Negative		Negative
Nucleosomes	Negative		Negative
Histone	Negative		Negative
smD1	Negative		Negative
PCNA	Negative		Negative
PO (RPP)	Negative		Negative

Table 3 shows the patient’s laboratory findings for specific autoimmune markers, all of which tested negative. The rheumatology profile revealed a normal anti-CCP IgG level (2.7 IU/mL), which is well below the positive threshold of 30 IU/mL, indicating no evidence of significant rheumatologic disease. 

**Table 3:** Autoimmune and rheumatology profile results

Test Name	Result	Unit	Reference Range
SS-A/Ro 60	Negative		Negative
SS-A/Ro 52	Negative		Negative
SS-B/La	Negative		Negative
CENP-B	Negative		Negative
Scl70	Negative		Negative
U1-snRNP	Negative		Negative
AMA-M2	Negative		Negative
JO-1	Negative		Negative
PM-Scl	Negative		Negative
MI-2	Negative		Negative
Ku	Negative		Negative
DF570	Negative		Negative
Anti-CCP IgG	2.7	IU/mL	<30

Table 4 displays elevated erythrocyte sedimentation rate (ESR) levels, indicating potential inflammation. C-reactive protein (CRP) levels were within the normal range, while Epstein-Barr virus (EBV) markers showed elevated IgG levels, suggesting a past EBV infection.

**Table 4:** Erythrocyte sedimentation rate (ESR), serology, and virology findings

Test Name		Result	Unit	Reference Range
Erythrocyte Sedimentation Rate (ESR)		120	mm/hr	1 - 20
C-Reactive Protein (CRP)		2.30	mg/dL	Less than 5.0 mg/dL: Negative More than 5.0 mg/dL: Positive
EBV - IgG		100	U/mL	Less than 20 U/mL: Negative More than 25 U/mL: Positive Between 20 - 25 U/mL: Equivocal
EBV - IgM		3.04	U/mL	Less than 20 U/mL: Negative More than 25 U/mL: Positive Between 20 - 25 U/mL: Equivocal

 Table 5 indicates significant abnormalities, including low red blood cell (RBC) count, hemoglobin (HGB) level, and hematocrit (HCT), consistent with severe anemia. Other abnormalities include reduced mean corpuscular volume (MCV), low mean corpuscular hemoglobin (MCH), and increased platelet count (PLT).

**Table 5:** Complete blood count (CBC) and blood film findings HCT: hematocrit; PCV: packed cell volume; MCV: mean corpuscular volume; MCH: mean corpuscular hemoglobin; MCHC: mean corpuscular hemoglobin concentration; RDW: red cell distribution width; CV: coefficient of variation; SD: standard deviation; MPV: mean platelet volume

Parameter	Result	Reference Range
WBC (Leukocytes)	6.14	4.0 - 10.0
NEU %	76.4	40 - 70
LYM %	18.0	20 - 45
MO %	5.0	2 - 10
EO %	0.4	0.5 - 5.0
BA %	0.1	0.0 - 1.0
RBC (Erythrocytes)	2.15 x10^12/L	4.0 - 5.5
HGB (Hemoglobin)	4.8 g/dL	12.0 - 16.0
HCT (PCV)	14.2 %	37.0 - 47.0
MCV	66.1 fL	80.0 - 100.0
MCH	22.4 pg	27.0 - 34.0
MCHC	33.8 g/dL	32.0 - 36.0
RDW - CV	16.7 %	11.0 - 14.0
RDW - SD	42.7 fL	-
Nucleated RBC %	0.02 /100WBC	0 - 0.0
Nucleated RBC #	0.001 x10^9/L	0 - 10,000.0
MPV	8.4 fL	7.0 - 10.0
PLT (Platelets)	469 x10^9/L	150.0 - 400.0

Table 6 highlights the repeat test two weeks after admission, revealing elevated ESR mm/hr, significantly higher than the previous value, indicating persistent inflammation. The tuberculosis screening test (TB-interferon-gamma release assay (IGRA)) result was below the threshold for infection (<0.35 IU/mL), ruling out active or latent tuberculosis infection.

**Table 6:** Follow-up inflammation profile and tuberculosis screening results. IGRA: interferon-gamma release assay

Test Name	Result	Reference Range
ESR - 1st hr	150	< 30 mm/hr
TB - IGRA	0.2 IU/mL	Noninfected: < 0.35; Indeterminate: 0.3; Infected: > 0.35

Table 7 summarizes the blot-line ANA test findings, showing positive results for several autoantibodies, including Ro52, Ro60, La, RNP-A, RNP-C, and RNP-68. The SmD antigen was borderline positive, and other markers were negative. The positive ANA findings suggest associations with autoimmune conditions such as Sjögren’s syndrome, systemic lupus erythematosus (SLE), and mixed connective tissue disease (MCTD).

**Table 7:** Blot-line ANA results and antibody evaluation ANA: antinuclear antibody

No.	Band Name	Position (mm)	Intensity (%)	Evaluation
1	IgG Control	-3.47	-	-
2	Ro-2	3.13	0.53	Negative
3	PL-7	5.16	0.53	Negative
4	PL-12	7.03	0.53	Negative
5	PmScl	10.16	0.53	Negative
6	CENP-A	12.11	0.53	Negative
7	CENP-B	14.14	0.53	Negative
8	Scl-70	16.09	0.53	Negative
9	Ro-52	19.64	33.79	Positive
10	Ro-60	21.67	39.02	Positive
11	La	23.62	43.99	Positive
12	RNP-A	25.65	26.39	Positive
13	RNP-C	28.62	20.47	Positive
14	RNP-68	30.48	36.49	Positive
15	SmB	32.51	8.15	Borderline
16	SmD	34.46	11.78	Positive
17	PCNA	37.42	2.05	Negative
18	dsDNA	41.49	2.55	Negative
19	Histone	43.43	2.03	Negative
20	Nucleosome	46.48	2.03	Negative
21	DFS70	49.61	5.89	Negative
22	M2	52.15	0.52	Negative
23	ANA	-	-	Positive

Table 8 presents test results for sarcoidosis-related markers and blood disease indicators. Angiotensin-converting enzyme (ACE) levels were elevated while complement 3 (C3) and complement 4 (C4) levels were within normal ranges, indicating no significant abnormalities related to complement activity.

**Table 8:** Sarcoidosis and blood disease marker findings

Test Name	Result		Unit	Reference Range
Angiotensin- onverting Enzyme (ACE)	34.58	U/L		< 40
Complement 3 (C3)	1.01	g/L		(0.9 - 1.8)
Complement 4 (C4)	0.10	g/L		(0.1 - 0.4)

Table 9 summarizes the results of additional biochemical and serological tests, highlighting values of kidney, liver, and immune function. The findings include elevated ESR and anti-dsDNA antibody levels, suggestive of inflammatory and autoimmune activity. Normal values in liver function tests and negative virology screening support the exclusion of viral involvement. This table shows elevated ANA and anti-DS DNA seven months from the start of her symptoms, roughly one month after her admission. Investigations shown in Tables 1-5 were done within the first week, while those in Table 6 were two weeks, and Tables 7-9 were almost one month after the admission.

**Table 9:** Additional biochemical and serological test findings SGOT: serum glutamic-oxaloacetic transaminase; AST: aspartate aminotransferase; SGPT: serum glutamic-pyruvic transaminase; ALT: alanine aminotransferase; ALK: alkaline; HIV-Ab: HIV antibody; HBsAg: hepatitis B surface antigen; HCV: hepatitis C

Test Parameter	Result	Reference Range
Erythrocyte Sedimentation Rate (ESR)	70 mm/hr	1-20 mm/hr
Anti-dsDNA Antibody	62 IU/mL	<0.8 IU/mL (Negative)
SGOT (AST)	24 U/L	Up to 40 U/L
SGPT (ALT)	30 U/L	Up to 45 U/L
Creatinine	0.23 mg/dL	0.6-1.1 mg/dL
Calcium (Total)	9 mg/dL	8.1-10.4 mg/dL
Potassium (K+)	3.70 mEq/L	3.6-5.5 mEq/L
Sodium (Na+)	143 mEq/L	135-155 mEq/L
Chloride	107 mEq/L	98-107 mEq/L
Bilirubin (Total)	0.60 mg/dL	Up to 1.0 mg/dL
Bilirubin (Direct)	0.39 mg/dL	Up to 0.25 mg/dL
ALK Phosphatase	50 U/L	Up to 115 U/L
HIV-Ab	Negative	Negative
HBsAg	Negative	Negative
HCV-Ab	Negative	Negative
ANA (Anti-Nuclear Antibody)	0.3 IU/mL	<0.8 IU/mL (Negative)

**Figure 1:**
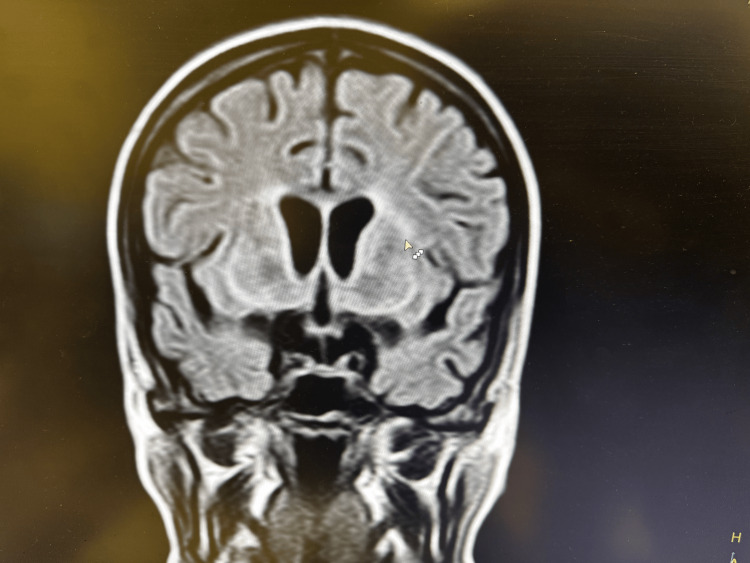
MRI of the brain showing perivasculitis as indicated by the small white arrow

A brain MRI was performed, showing small perivascular lesions suggestive of vasculitis changes (Figure 1). The cervico-dorsal MRI was negative.

**Figure 2:**
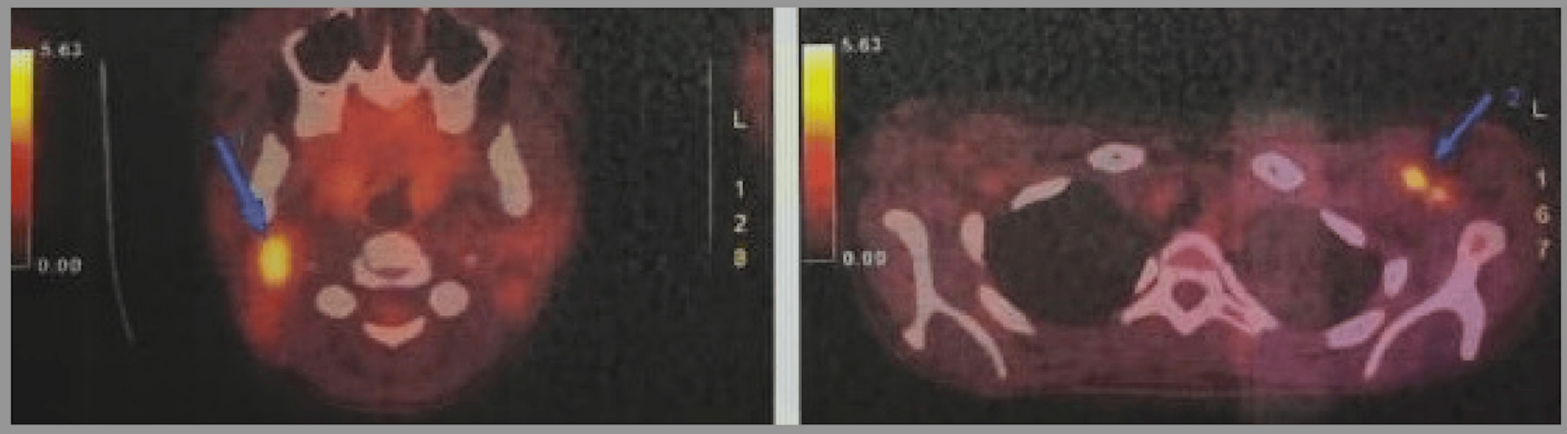
Hypermetabolic active lymphadenopathy in cervical and axillary regions as indicated by the blue arrow

Whole body scan (PET scan) demonstrated hypermetabolic activity in cervical, axillary, and supraclavicular lymphadenopathy. Diffuse fluorodeoxyglucose (FDG) uptake in the spleen was considered reactive (Figure 2).

Cervical lymph node biopsy reported findings of subacute necrotizing lymphadenitis; no malignancy could be identified in examined material. Unfortunately, biopsy pictures were unavailable for publication. 

**Figure 3:**
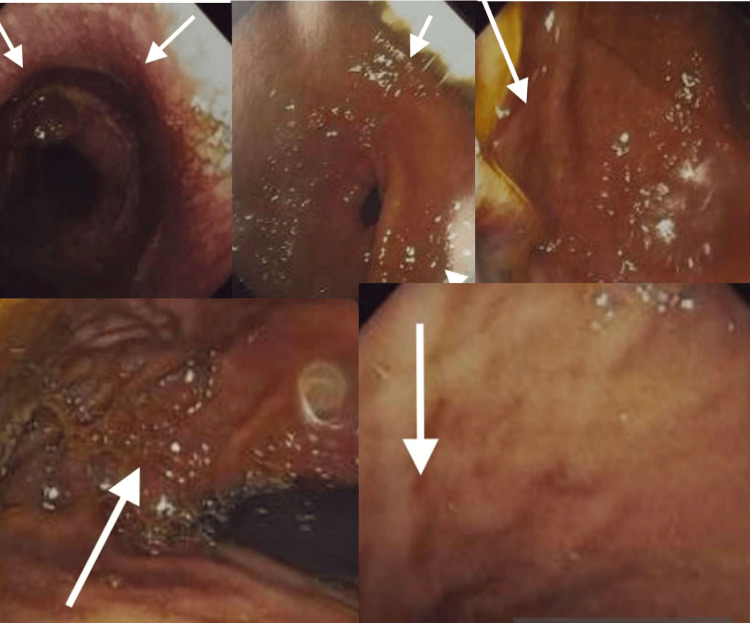
Endoscopic views of inflamed gastric mucosa with signs of gastritis

Upper gastrointestinal endoscopy revealed mild erythematous gastritis and lax cardia (Figure 3).

She also reported a negative Mantoux test and clear sputum done a month before her admission in a different clinical setup. Electroencephalogram (EEG) at the time of admission in our unit showed epileptiform discharge with generalized slowness. No pictures were available, and the EEG was not repeated. Following an extensive diagnostic evaluation and after systematically excluding infectious, malignant, and other differential causes, a diagnosis of biopsy-confirmed Kikuchi-Fujimoto disease was established. The patient was in remission on symptomatic treatment with fever subsiding and partial improvement in fatigue and loss of appetite; however, difficulty in walking and loss of power in hands remained progressive, indicating a relapse. Upon relapse, the clinical presentation raised strong suspicion of an underlying systemic autoimmune disorder, prompting further immunological assessment. This revealed positive antinuclear antibodies (ANA), anti-dsDNA antibodies, and hypocomplementemia with reduced C3 and C4 levels, findings highly suggestive of an evolving SLE flare. The patient was initiated on intravenous methylprednisolone (500 mg daily for five days), followed by a gradual transition to oral prednisolone (0.5 mg/kg/day) and hydroxychloroquine (5 mg/kg/day), in accordance with established international guidelines for lupus management. This regimen led to a significant reduction in fever and improvement in mobility. Subsequently, she received cyclophosphamide (Endoxan) infusions, started in the first week of her admission to our unit and still ongoing, which resulted in a dramatic improvement in her walking ability, mood stabilization, and cessation of seizures. She had not been on any antiepileptic drugs prior to admission, but in our center, the patient was kept on an antiepileptic levetiracetam tablet 500 mg twice daily.

## Discussion

KFD is a self-limiting necrotizing lymphadenitis, most often seen in younger female patients. The leading clinical feature is cervical lymphadenopathy, which affects nearly all individuals diagnosed with KFD, often accompanied by fever in a significant proportion of cases. Other symptoms, including rash, joint pains, fatigue, and organomegaly, have also been described, though they occur much less frequently [6]. A definitive diagnosis is made through histopathological assessment of tissue obtained via excisional lymph node biopsy, which remains the gold standard, though ultrasound-guided fine needle aspiration has been employed in select cases when excision is impractical [7]. Characteristically, histology demonstrates paracortical necrosis accompanied by histiocytic infiltration, providing a distinct pathological signature.

The link between KFD and SLE is well-recognized, with cases reported where KFD either precedes, coincides with, or follows the development of SLE. In addition to SLE, conditions such as adult-onset Still’s disease, SARS-CoV-2 infection, and Sjögren’s syndrome have also been described alongside KFD, suggesting that immune dysregulation may play a critical role in its pathogenesis [8-10]. Given the overlapping features between autoimmune and infectious processes, patients presenting with fever and lymphadenopathy should undergo a broad infectious screen, including tests for Epstein-Barr virus, cytomegalovirus, HIV, toxoplasmosis, Yersinia enterocolitica, and Bartonella henselae to rule out infectious mimics [4].

While KFD and SLE share some clinical and immunological overlap, like lymph node histology and immune activation patterns, key differences help differentiate the two conditions. KFD tends to lack ANA positivity, remains limited to the lymph nodes, and resolves spontaneously within a few months [11]. In contrast, SLE is characterized by high ANA positivity, multi-system involvement, and typically requires long-term immunosuppressive therapy to control disease activity.

In this patient, the initial diagnostic workup considered a range of possible causes, including tuberculosis, lymphoma, viral infections, and sarcoidosis. Tuberculosis was effectively ruled out through a combination of negative Mantoux testing, clear sputum examination, and absence of granulomas on lymph node biopsy. Lymphoma was excluded following cross-sectional imaging and comprehensive histological review. Viral panels, including Epstein-Barr virus, retroviruses, and rickettsial infections, were all negative. Radiological findings, specifically the absence of hilar or mediastinal lymphadenopathy, also made sarcoidosis unlikely. Ultimately, the diagnosis of KFD was confirmed through the pathognomonic histological findings in the lymph node specimen.

Since KFD generally resolves on its own, treatment is usually supportive, with symptoms improving within one to four months [11]. For patients with significant symptom burden, glucocorticoids can be offered. In refractory or severe recurrent cases, combining high-dose glucocorticoids with intravenous immunoglobulin (IVIG) may be beneficial [12]. Cases of relapsing KFD have also been treated with hydroxychloroquine monotherapy, or in some situations, a combination of glucocorticoids and interleukin-1 inhibitors, such as anakinra, particularly when steroid resistance develops [13,14].

## Conclusions

Kikuchi-Fujimoto Disease (KFD) is generally recognized as a benign and self-limiting disorder, though recurrent episodes have been documented. The presence of positive fluorescent antinuclear antibodies (ANA) has been linked to a higher recurrence risk. While KFD is not always a prelude to systemic lupus erythematosus (SLE), its concurrent occurrence with SLE presents diagnostic complexities. Clinicians must remain aware of this potential overlap to minimize diagnostic errors and prevent unnecessary or inappropriate treatment.

A comprehensive diagnostic approach is essential to rule out infectious, autoimmune, and malignant causes. Although supportive care suffices in most cases, patients with persistent or recurrent symptoms may benefit from targeted therapies. Furthermore, long-term follow-up is advised to enable early identification of evolving autoimmune or systemic disorders.
